# Antioxidant Potential of Curcumin—A Meta-Analysis of Randomized Clinical Trials

**DOI:** 10.3390/antiox9111092

**Published:** 2020-11-06

**Authors:** Karolina Jakubczyk, Aleksandra Drużga, Janda Katarzyna, Karolina Skonieczna-Żydecka

**Affiliations:** Department of Human Nutrition and Metabolomics, Pomeranian Medical University in Szczecin, 24 Broniewskiego Street, 71-460 Szczecin, Poland; karjak@pum.edu.pl (K.J.); olaaad@gmail.com (A.D.); karzyd@pum.edu.pl (K.S.-Ż.)

**Keywords:** curcumin, polyphenol, antioxidant, oxidative stress, turmeric, Indian saffron, meta-analysis

## Abstract

Background: Antioxidant potential is defined as the ability to neutralize oxygen free radicals that are generated in excess due to environmental influences. The body’s defense mechanisms often require support in preventing the effects of oxidative stress. The literature data suggest that curcumin has antioxidant activity that can significantly reduce oxidative stress levels. The aim was to assess the impact of curcumin on oxidative stress markers. Methods: PubMed and Embase were searched from database inception until 27 September 2019 for randomized clinical trials in >20 patients treated with curcumin supplements and randomized to placebo/no intervention/physical activity to verify the antioxidant potential of curcumin. Results: Four studies were included in the meta-analysis, three of which were double-blind and one single-blind. A total of 308 participants took part in the research. A total of 40% of the respondents were men. The average age of participants was 27.60 ± 3.79 years. The average supplementation time was 67 days and the average dose of curcumin administered was 645 mg/24 h. Curcumin significantly increased total antioxidant capacity (TAC) (SMD = 2.696, Z = 2.003, CI = 95%, *p* = 0.045) and had a tendency to decrease malondialdehyde (MDA) concentration (SMD = −1.579, Z = −1.714, CI = 95%, *p* = 0.086). Conclusions: Pure curcumin has the potential to reduce MDA concentration and increase total antioxidant capacity.

## 1. Introduction

Oxidative stress is an imbalance in the production of reactive oxygen species (ROS), and the ability to neutralize them and repair damage. Under physiological conditions, cells are maintained in a reducing environment, provided by endogenous antioxidants or antioxidant enzymes. Increased levels of oxidative stress have consequences such as lipid, protein, and nucleic acid modifications, which ultimately lead to a worsening of mitochondrial function and cell death [1,2]. In turn, so many dysfunctions lead to many disorders, among others neurodegenerative diseases (Alzheimer’s and Parkinson’s disease), atherosclerosis, obesity, or simply aging [1,2,3,4]. Searching for natural substances with high antioxidant potential seems to be an effective preventive measure in free radical-linked phenotypes.

Curcumin (C_21_H_2_OO_6_) is a lipophilic substance of a polyphenol nature. It is obtained from rhizomes of turmeric (*Curcuma longa* L.)—a plant belonging to the ginger family, commonly known as Indian saffron or turmeric. Turmeric is an important component of curry spice, which gives a characteristic yellow color and is also used as a dye. Turmeric powder obtained during the drying process contains curcuminoids. They consist of 77% curcumin, which does not dissolve in acid or neutral water. Instead, it is soluble in acetone, methanol, and ethanol [5,6,7,8].

Curcumin, through its chemical structure and the presence of hydroxyl and methoxy groups, is attributed to many properties, in particular antioxidant, antimicrobial, anti-inflammatory, anti-angiogenic, and antimutagenic ones [5,6,9,10,11,12]. Curcumin is a potentially useful additive supporting the treatment of irritable bowel syndrome (IBS) due to its unique action, especially antioxidant and anti-inflammatory, and the ability to modulate the intestinal microbiota [13]. In addition, due to its ability to interact with various molecular targets, curcumin inhibits inflammatory cell proliferation and angiogenesis, as well as acts chemopreventively [5,6,9,10,11,12]. These properties are associated with the regulation of proinflammatory cytokines, nitric oxide synthase (iNOS) enzymes, cyclooxygenase-2 (COX-2), lipoxygenase, xanthine oxidase, and reduction in malondialdehyde (MDA) [5,6,9,10,11,12,14].

Research suggests that curcumin may be an effective antioxidant that minimizes the effects of oxidative stress. By being able to interact with various molecular mechanisms, it reduces the level of oxidative stress, which is related to, among others, the ability to chelate heavy metals or regulate the activity of many enzymes. In addition, the Food and Drug Administration (FDA) confirmed that curcumin is a compound generally recognized as safe. There is one systematic review combined with meta-analysis, performed by Alizadeh and Kheiouri, describing an intervention of curcumin and piperine, which increased the bioavailability of curcumin and their effect on antioxidant potential [15]. Therefore, the purpose of this meta-analysis is to assess the antioxidant potential of pure curcumin. The meta-analysis was based on randomized clinical trials (RCTs) with regard to the antioxidant potential of pure curcumin.

## 2. Materials and Methods

### 2.1. Search Strategy, Inclusion Criteria

At least two independent authors (KJ, AD, KSZ) searched PubMed/MEDLINE/Embase from database inception until 27 August 2019 without language restrictions to identify RCTs assessing pure curcumin’s antioxidant potential compared to control interventions

The following search string in PubMed was used: human AND (curcumin OR curcumine OR turmeric yellow) AND (placebo OR monotherapy OR no intervention OR physical activity) AND (antioxidant OR antioxidant agent OR antioxidant nutrient OR antioxidants OR antioxidation agent OR antioxidation product OR antioxidative OR antioxidant OR phenolic antioxidant).

In Embase, the search string was: “human”/exp AND (“curcumin”/exp OR “curcumin” OR “curcumine” OR “turmeric yellow”) AND (“placebo”/exp OR “placebo”) AND (“antioxidant”/exp OR “antioxidant” OR “antioxidant agent” OR “antioxidant nutrient” OR “antioxidants” OR “antioxidation agent” OR “antioxidation product” OR “antioxidative” OR “antioxidant” OR “phenolic antioxidant”).

We utilized the following inclusion criteria: (i) randomized controlled trial, (ii) >20 randomized participants, (iii) treatment with pure curcumin supplements at any dose, (iv) randomization to curcumin vs. no intervention/physical activity, and (v) available meta-analyzable endpoint data (preferred)/change score on any of parameters linked to oxidative stress. Exclusion criteria were: (i) studies with persons randomized on more than curcumin supplementation at once (i.e., cointervention, like curcumin and any other biocompound with antioxidant potential, curcumin and a drug), (ii) curcuminoids as intervention substances. 

### 2.2. Data Abstraction

Data for country in which the study was conducted, information about the sponsors, type of blindness, duration of the study, and main purpose of the study, as well as the name of the preparation used during therapy were extracted. During data abstraction, detailed data on the studied population were looked for, i.e., the average age and standard deviation of studied persons, the number and percentage of men participating in the study, as well as the number of people randomized to the study. Data extraction was performed based on the guidelines contained in the Preferred Reporting Items for Systematic Reviews and Meta-Analyses (PRISMA) protocol. If data were missing, authors were contacted via email to ask for additional information. Inconsistencies were resolved by consensus with the corresponding author being involved. Data from charts and figures were extracted by means of WebPlotDigitizer software (https://automeris.io/WebPlotDigitizer/). In order to detect the risk of bias, the Cochrane Collaboration’s tool for assessing risk of bias was used. 

### 2.3. Outcomes

The main result evaluated in the meta-analysis was the effect of curcumin on the parameters of oxidative stress parameters. Co-primary outcomes were: Superoxide dismutase (SOD), Glutathione peroxidase (GPx), catalase (CAT), glutathione reductase (GR/GSR), glutathione *S*-transferase (GST), Paraxonase 1 (PON1), eosinophil peroxidase (EPO), total oxidant capacity (TOC), lipid hydroperoxides (LHP), lipopolysaccharide (LPS), protein sulfhydryl (PSH), malondialdehyde (MDA), glutathione activity (GSH), total antioxidant status (TAS), ROS, superoxidative anion radical (O^2−^), nuclear factor erythroid 2-related factor 2 (Nrf2), oxygen radical absorbance capacity (ORAC), 2, 2′-azinobis (3-ethylbenzothiazoline-6-sulfonic acid) (ABTS), 2,2-diphenyl-1-picrylhydrazyl (DPPH), ferric reducing antioxidant potential (FRAP), (cupric-reducing antioxidant capacity (CUPRAC), and pro-oxidant–antioxidant balance (PAB).

### 2.4. Statistical Analysis

The statistical analyses were conducted using Comprehensive Meta-Analysis software (version 3.3.070; http://www.meta-analysis.com). The between-study variance was estimated using the method of moments (DerSimonian and Laird) and the assumption of homogeneity in effects was tested using the Q statistic with a k − 1 degree of freedom (k—the number of studies). Pooled standardized mean difference (SMD) in change score/endpoint scores was used to analyze group differences in the case of continuous variables. A two-tailed Z test was used to test the null hypothesis that the summary effect was zero.

## 3. Results

### 3.1. Search Results

The first search in the PubMed and Embase databases resulted in 759 hits. Among them, 639 studies were excluded as duplicates and/or after evaluation at the title/abstract level. After excluding 639 studies, 120 full-text articles were eventually reviewed, 116 of which were excluded due to the failure to meet previously established inclusion criteria.

**Figure 1:**
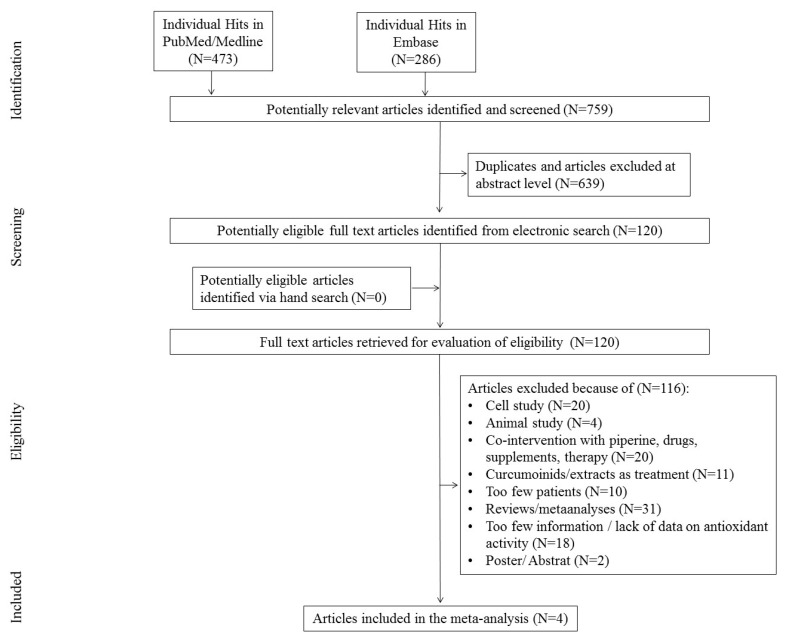
Study flow chart.

The main reasons for exclusion were: review/meta-analyses (N = 31), too little information, lack of information on antioxidant activity (N = 28), co-interaction with piperine, drugs, and other supplements (N = 15), cellular study (N = 20), animal studies (N = 4), curcuminoids used in therapy (N = 6), too few participants (N = 10), and poster/abstract (N = 2). Finally, four studies were included in the meta-analysis. The scheme of searching databases is included in Figure 1.

### 3.2. Study Characteristics

Four studies included between 2017 and 2019 were included in the meta-analysis. The aim of all studies was to assess the effect of curcumin administration on oxidative stress parameters. All studies were conducted in Iran. The research was financed with university funds. Three double-blind and one single-blind study were included in the meta-analysis. The detailed characteristics of the studies included in the meta-analysis are presented in Table 1.

### 3.3. The Impact of Curcumin on Oxidative Stress-Related Outcomes

**Figure 2:**
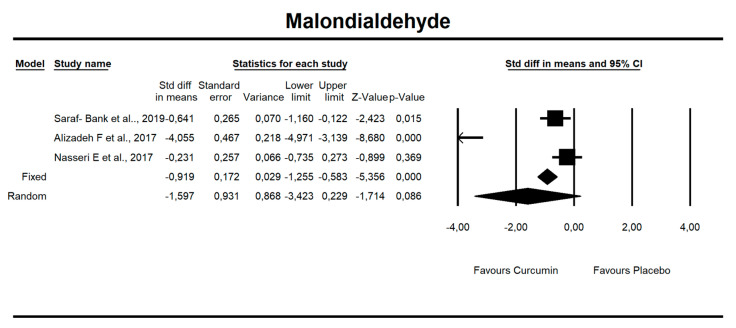
An effect size standardized mean difference, for MDA at endpoint in persons taking curcumin vs. controls (endpoint data). Q = 53.330, df(Q) = 2, *p* = 0.000, I-squared = 96.250.

**Figure 3:**
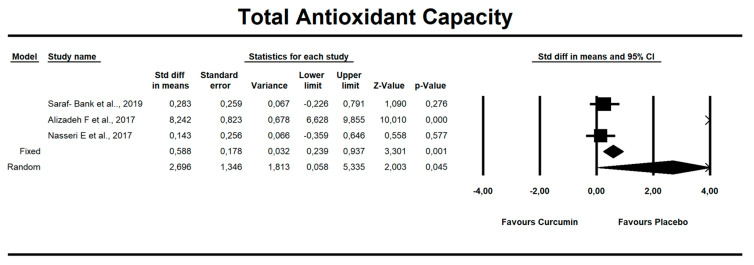
An effect size standardized mean difference, for TAC at endpoint in persons taking curcumin vs. controls (endpoint data). Q = 90.804, df(Q) = 2, *p* = 0.000, I-squared = 97.797.

Despite the number of parameters abstracted from studies, we were able to meta-analyze two parameters only, i.e., MDA concentration and TAC. It has been noted that there is a tendency to reduce MDA concentration after curcumin (SMD = −1.579, Z = −1.714, CI = 95%, *p* = 0.086). Similarly, it was proven that the TAC is significantly higher after application of the said compound (SMD = 2.696, Z = 2.003, CI = 95%, *p* = 0.045). The results are presented in Figure 2 and Figure 3. Given the heterogeneity of the studies qualified for the meta-analysis, a variable model was also adopted, although the calculations were also made in a fixed model.

It has been established that there is a statistical trend that indicates that curcumin intake results in a decrease in MDA value by 1.5 standardized units (SMD = −1.597, CI = 95%, *p* value = 0.086.

Curcumin administration resulted in significant increase in TAC parameter (SMD = 2.696, Z = 2.003, CI = 95%, *p* = 0.045). The other oxidative-stress linked parameters that were abstracted from the studies are shown in Supplementary Table S1.

### 3.4. Risk of Bias Assessment

The bias analysis showed that the two studies were of low quality and received less than 5 points in the ROB evaluation [16,17]. In the last two [10,18], the number of points was 5. The average number of points in all studies is 4. The results of the bias risk analysis are presented in Table 2.

**Figure 4:**
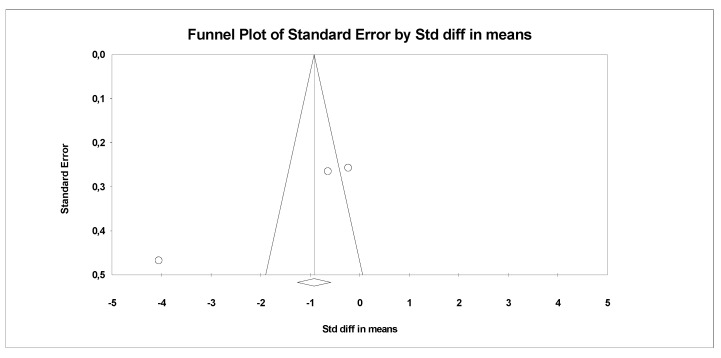
Funnel plot for endpoint MDA (SMD) in present meta-analysis.

**Figure 5:**
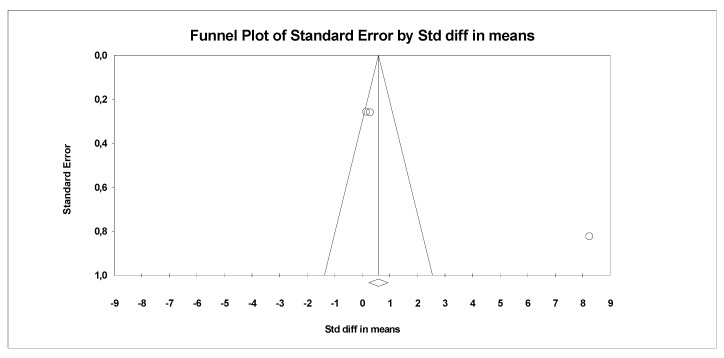
Funnel plot for endpoint TCA (SMD) in present meta-analysis.

In the last stage of research, an analysis of publication bias was performed. To this end, funnel charts were made and Egger’s test was performed (Figure 4 and Figure 5). No publication bias was detected for MDA: t = 9.81, *p* = 0.06. Alternative results were obtained for TACs: t = 36.05, *p* = 0.01. All parameters related to oxidative stress are placed in Supplementary Data (Table S1).

## 4. Discussion

Curcumin, a bioactive polyphenol, is a common ingredient in Indian cuisine. It is also popular in South Asia and the Middle East; however, it is also increasingly used in European countries [19,20]. Curcumin acts on various molecular pathways, and it also has great potential in the fight against chronic diseases due to its biological activity [21,22]. However, curcumin’s antioxidant properties are considered as key ones [22]. 

Oxidative stress, which is characterized by an imbalance between the production and elimination of ROS, is associated with aging processes and many chronic diseases [1,2]. However, due to the very short half-life of ROS, it is impossible to measure their levels directly. Hence, measurements of changes in MDA, TAC, CAT, GPx, and SOD levels are carried out, which are considered significant manifestations of oxidative stress [23]. For instance, high initial levels of MDA concentration are correlated with the occurrence of excess free radicals, thus making dialdehyde an excellent marker of oxidative stress. Many studies indicate curcumin as an agent having the ability to reduce serum MDA levels as well as increase SOD and GPx activity [24,25,26]. Clinical trials regarding the aforementioned curcumin effects report promising effects. However, often these studies include curcuminoids with piperine or extracts that are a mixture of different compounds [27,28,29,30]. 

This meta-analysis was conducted in a group of 308 people, with a predominance of women (60%). In all enrolled studies, curcumin was administered daily, for between 42 and 84 days, at a dose of 80 to 1000 mg per day. In three studies, tablets were used as a form of curcumin administration, while in one study, curcumin was administered as nanoparticles. In the study by Saraf-Bank et al. [18], a group of obese teenagers was evaluated. During the 70-day study, girls were given 500 mg curcumin tablets. A relatively high baseline MDA of 143.36 μM was reported in the curcumin group. This may be related to obesity and teenage age. It was elegantly demonstrated that obesity is a predictor of increased levels of oxidative stress markers, including MDA [31]. However, after curcumin supplementation, the MDA level decreased significantly and was equal to 73.78 μM (*p* = 0.009). In a study by Alizadeh et al. [10], the effect of reducing the level of oxidative stress markers and increasing antioxidant capacity seems to be closely related to the mechanism of direct action of curcumin by cleansing the body of free radicals, as well as increasing the activity of antioxidant enzymes [10]. The 84-day study of Nasseri et al. [16], in which 500 g of curcumin as tablets was administered twice daily to patients suffering from β-thalassemia, gave similar results. It is worth mentioning that the initial level of antioxidant enzymes, e.g., catalase, as well as the total antioxidant capacity, were reduced. The likely cause is the tendency to increase the need to neutralize oxidative stress in patients with β-thalassemia. Similarly to the obese, these persons are characterized by elevated levels of MDA as an indicator of lipid peroxidation [16,32]. The level of MDA serum concentration after curcumin intervention decreased (*p* = 0.002, *p* < 0.001). In contrast, the marker assessing the total antioxidant capacity increased, compared to the initial values (*p* = 0.005). In turn, the catalase and TAC levels remained unchanged between the groups (*p* > 0.05). A study by Ghazimoradi et al. showed that 42-day curcumin supplementation at a dose of 1 g daily significantly improved the balance of pro-oxidant–antioxidant in the intervention group [17].

The results of the present meta-analytical study prove that curcumin has the potential to reduce MDA concentration (SMD = −1.579, Z = −1.714, CI = 95%, *p* = 0.086). Similarly, it was proved that TAC is significantly higher after application of the said compound (SMD = 2.696, Z = 2.003, CI = 95%, *p* = 0.045), which confirms the antioxidant properties of this substance. This is consistent with the results of the original research and the meta-analyses carried out so far. Alizadeh M. and Kheiouri S. performed a systematic review combined with meta-analysis, including intervention of both curcumin and piperine, which increases the bioavailability of curcumin [15]. The study aimed to analyze the effect of curcumin supplementation on MDA concentration and levels of antioxidant markers in the study group compared to the placebo group. The authors analyzed the topic in the PubMed, Embase, Cochrane Central, Scopus, and Google Scholar databases. However, the meta-analysis was carried out using RevMan (version 5.3). Review of the results in the databases, based on the phrases determined by the authors, brought 124 papers. Finally, considering the inclusion and exclusion criteria, a meta-analysis was performed on a group of 17 papers. Parameters such as MDA, TAC, SOD, CAT, GPx, and GSH were measured. Curcumin was administered in various doses ranging from 80 mg to 4 g per day. The analysis indicates the effectiveness of curcumin in reducing MDA levels and the potential to improve antioxidant levels. Reduction in oxidative stress depended on the duration of treatment and the curcumin dose administered, as well as the presence of piperine [15]. 

Similar results regarding MDA and TAC are shown in the study by Judaki et al. [33]. The aim of the study was the effect of curcumin at the level of oxidative stress markers and the symptoms of histopathological mucositis in patients affected by *Helicobacter pylori* infection. Patients were divided into a control group undergoing triple therapy, i.e., a fixed dose of a complex of three drugs (omeprazole 20 mg, amoxicillin 1000 mg, metronidazole 800 mg—each administered orally, twice a day) and an intervention group in which a triple therapy and a dose 700 mg curcumin three times a day were given [33,34]. MDA concentration in the intervention group decreased significantly. This change was also statistically significant in relation to the control group. The results also show the potential of curcumin to increase overall antioxidant capacity [33]. Previous studies have shown that patients affected by *H. pylori* infection tend to have increased ROS production and increased MDA levels, which results in oxidative damage at the DNA level and, as a result, can initiate a carcinogenesis process. [35]. The pattern observed in the results is explained by the antioxidant properties of curcumin [36] and the ability to inhibit growth factor and at the same time, reduce the level of nuclear factor (NF-kB) [35,37]. This study, however, differs from the co-drug interactions included in the present meta-analysis. It is therefore possible that despite similar observations, the result could be affected by interfering factors in the form of drugs administered in triple therapy.

Research conducted by Panahi et al. [27] evaluated the effectiveness of curcuminoid + piperine administration in alleviating systemic oxidative stress and improving quality of life in people with chronic pulmonary complications. The observations obtained by the authors prove the high effectiveness of the complex in reducing the level of MDA concentration. The addition of piperine was used to increase the bioavailability of curcuminoids [38,39]. However, this could have had a significant impact on the final results. According to the Mao et al. study [40], piperine alone, even without the addition of curcuminoids, has the potential to reduce MDA levels [40,41]. 

In a study by Wongcharoen et al. [42], similar results were obtained, confirming the potential of curcumin to lower MDA concentration. The aim of the study was to evaluate whether curcuminoids prevent myocardial infarction after coronary artery bypass surgery compared to placebo. Adequate myocardial protection after CABG (coronary artery bypass grafting) is crucial in myocardial infarction [43]. Curcuminoids were administered in the form of tablets, in an amount of 4000 mg per day. The tablet contained desmethoxycurcumin, bisdemethoxycurcumin, and curcumin in proportions of 0.6:0.3:1.0, respectively. In the intervention group a decrease in MDA level was observed (−5.7 ± 1.5 mmol/mL, *p* < 0.001) [42]. The mechanism is explained by the antioxidant properties of curcuminoids, due to which they have the ability to inhibit inflammatory mediators [14,42]. The results obtained in the study may have been affected by the use of curcuminoids rather than pure curcumin, which could have enhanced the effect.

Observations contradicting the antioxidant benefits of curcumin come from a randomized, double-blind study by Hodaei H. et al. [44]. The main purpose of the study was to assess the effect of curcumin supplementation on anthropometric parameters, oxidative stress parameters, and glycemic control in overweight and type II diabetes patients. Patients were given 1500 mg curcumin or placebo capsules, respectively, three times a day for 10 weeks. Analysis of the results did not show significant changes in MDA and TAC levels [44]. This may be due to concomitant type 2 diabetes mellitus and hyperglycemia occurring in patients, which potentially increases the production of free radicals and the level of oxidative stress [45,46]. In addition, the study lasted 10 weeks, which may be too short a time for curcumin to influence the analyzed factors enough to show statistically significant differences in the analyzed time period [44]. 

## 5. Conclusions

In summary, this meta-analysis has shown that pure curcumin has antioxidant properties. It reduces the concentration of malondialdehyde (MDA) in serum and has potential to increase the total antioxidant potential (TAC). The action of curcumin on markers of oxidative stress is associated with its properties directed to the removal of reactive oxygen and nitrogen, metal chelation, and regulation of numerous enzymes. Curcumin has therefore been proven to be an antioxidant. Further research, however, is necessary due to the still too few studies assessing pure curcumin (not curcuminoids, without the addition of enhancers, such as piperine) and limitations in the form of a diverse study population affected by various diseases, which may significantly affect the results.

## Figures and Tables

**Table 1:** Study characteristics.

Study Description				Intervention			Persons Analyzed		
Reference/Year/Country/Sponsorship	Blinding/Crossover (Y/N)/Multi-Arm > 2	Focus on	ROB	The Form of Supplement	Curcumin Dose/(mg/day)	Duration of Administration (days)/Comparator	N Total Randomized/Analyzed	Age Years (Mean ± SD)	Males (*n*/%)
Saraf-Bank/2019/Iran/Academia	SB/N/N	MDA, TCA	5	Pills	500	70/placebo	60/60	16.001 ± 1.64	0/0
Alizadeh/2017/Iran/Academia	DB/N/N	MDA, TCA	5	Nanomicelle	80	70/placebo	60/56	30.27 ± 4.00	60/100
Ghazimoradi/2017/Iran/Academia	DB/N/N	PAB	4	Pills	1000	42/placebo	120/109	38.05 ± 2.89	19/17.43
Nasseri/2017/Iran/Academia	DB/N/N	MDA, TAC, Catalase	2	Pills	1000	84/placebo	68/61	26.80 ± 6.64	26/42.62

DB—double blind; SB—single blind; N—no; Y—yes; NA—not applicable; ROB—risk of bias; SD—standard deviation; MDA—malondialdehyde; TAC—total antioxidant capacity; PAB—pro-oxidant–antioxidant balance.

**Table 2:** Risk of bias (ROB).

No.	Reference/Year/Country/Sponsorship	Random Sequence Generation (Selection Bias)	Allocation Concealment (Selection Bias)	Blinding of Participants and Personnel (Performance Bias)	Blinding of Outcome Assessment (Detection Bias)	Incomplete Outcome Data Addressed (Attrition Bias)	Selective Reporting (Reporting Bias)	Other Bias	No. of Low ROB Assessments
1	Saraf- Bank/2019/Iran/Academia	L	L	L	?	L	L	?	5
2	Alizadeh/2017/Iran/Academia	L	L	L	?	L	L	?	5
3	Ghazimoradi/2017/Iran/Academia	L	L	?	?	L	L	?	4
4	Nasseri/2017/Iran/Academia	H	H	?	?	L	L	?	2

L—low risk of bias; H—high risk of bias; ?—unclear risk of bias.

## Supplementary Materials

The following are available online at https://www.mdpi.com/2076-3921/9/11/1092/s1, Table S1 Additional parameters related to oxidative stress.
