# Supplementary material for: Antioxidant Potential of Curcumin—A Meta-Analysis of Randomized Clinical Trials
Source: Antioxidants (Basel). 2020 Nov 6;9(11):1092. doi: 10.3390/antiox9111092 (PMC7694612; doi:10.3390/antiox9111092)
Supplement: Supplementary file 1 [file antioxidants-09-01092-s001.pdf]

Ref	POB					
	Treatment group			Control group		
	mean	sd	n	mean	sd	n
Feguri et al., 2017, Brazil	353.57	355.43	14	204.64	99.78	14
Savluk et al., 2017a, Turkey	837	501	38	785	435	39
