# Supplementary material for: Antioxidant Potential of Curcumin—A Meta-Analysis of Randomized Clinical Trials
Source: Antioxidants (Basel). 2020 Nov 6;9(11):1092. doi: 10.3390/antiox9111092 (PMC7694612; doi:10.3390/antiox9111092)
Supplement: Supplementary file 2 [file antioxidants-09-01092-s002.pdf]

Reference	Trial duration (days)	Curcumin dose [mg]	Comparator	Malondialdehyde (MDA) baseline						
					resveratrol			PBO		
				unit	Ø	±	n	Ø	±	n
Saraf-Bank/2019/Iran/Academia	70	500	PBO	µM	143.36	81.69	30	121.4	60.13	30
Alizadeh/2017/Iran/Academia	70	80	PBO	µmol/l	0.96	0.09	28	1.05	0.1	28
Ghazimoradi/2017/Iran/Academia	42	1000	PBO							
Nasseri/2017/Iran/Academia	84	1000	PBO	µmol/l	41.03	14.96	31	38.7	12.24	30

^quartile

Malondialdehyde (MDA) endopint							Plasma total antioxidant capacity (TAC)							Plasma total ar	
	resveratrol			PBO				resveratrol			PBO				resv
unit	Ø	±	n	Ø	±	n	unit	Ø	±	n	Ø	±	n	unit	Ø
μM	73.78	51.5	30	103.23	39.55	30	mM	0.22	0.03	30	0.23	0.04	30	mM	0.23
μmol /l	0.73	0.07	28	1.08	0.1	28	μmol/l	1.24	0.07	28	1.36	0.08	28	μmol/l	1.95
μmol /l	37.12	11.7	31	39.9	12.37	30	μmol/L	187.09	82.26	31	191.46	76.16	30	μmol/L	207.96

tioxidant capacity (TAC)					Antioxidant balance (PAB) baseline							Antioxidant balance (PAB) e				
eratrol		PBO				resveratrol			PBO				resveratrol			
**±**	**n**	**Ø**	**±**	**n**	**unit**	**Ø**	**±**	**n**	**Ø**	**±**	**n**	**unit**	**Ø**	**±**	**n**	**Ø**
0.04	30	0.22	0.03	30												
0.05	28	1.35	0.09	28												
					HK	92.29	66.10-126.02^	36	122.94	66.44-159-96^	36	HK	142.14	98.05-184.41^	36	110.43
91.72	31	196.1	72.57	30												

ndpoint		Catalase (CAT) baseline							Catalase (CAT) endpoint						
PBO			resveratrol			PBO				resveratrol			PBO		
±	n	unit	Ø	±	n	Ø	±	n	unit	Ø	±	n	Ø	±	n
87.89-128.36^	36														
		μ/mL	5.68	3.95	31	6.2	3.73	30	μ/mL	5.81	3.76	31	6.01	3.87	30
